# Hormone refractory carcinoma prostate with peritoneal metastases and malignant ascites without skeletal involvement: A case report and review of literature

**DOI:** 10.4103/0970-1591.65405

**Published:** 2010

**Authors:** Samuel P. Benedict, Manish Ahuja, Kim J. Mammen

**Affiliations:** Department of Urology, Christian Medical College and Hospital, Ludhiana - 141 008, Punjab, India

**Keywords:** Metastasis, peritoneum, prostate cancer

## Abstract

Peritoneal carcinomatosis is rare in prostate cancer especially in the absence of skeletal or other visceral metastases. We report a case of hormone refractory adenocarcinoma prostate presenting with only peritoneal metastases and massive malignant ascites. Palliation with docetaxel based cytotoxic chemotherapy resulted in clinical improvement of refractory ascites decreasing respiratory embarrassment and thereby improving the quality of life.

## INTRODUCTION

Peritoneal metastasis is a rare finding in prostate cancer especially in the absence of skeletal or other visceral metastases. We report a case of hormone refractory adenocarcinoma prostate presenting with only peritoneal metastases and massive malignant ascites. A brief review of the pattern of prostate cancer metastasis, factors influencing spread and management strategies are discussed.

## CASE REPORT

A 67-year-old gentleman, a diagnosed case of adenocarcinoma prostate, treated with complete androgen ablation including bilateral orchiectomy and bicalutamide for two years presented with progressive abdominal distension and breathing difficulty of two weeks duration. Physical examination revealed a cachectic patient with massive ascites causing respiratory embarrassment, a hard nodular prostate, and a percutaneous nephrostomy on the left side.

A review of the medical history showed that the patient had undergone TURP for lower urinary tract symptoms two years earlier and the histopathology of the prostate specimen had revealed adenocarcinoma of the prostate (Gleason score 8; 4 + 4). The patient's baseline serum PSA level was 36.4 ng/ml; however, a metastatic workup was negative. Following androgen ablation, his serum PSA level reached a nadir of 1.14 ng/ml in 12 months time. However, he needed multiple endoscopic procedures for recurrent stricture of the membranous urethra. Twenty two months after initiation of hormonal therapy, he developed abdominal distension and malaise. His serum PSA level was 14.3 ng/ml. Obstructive uropathy was diagnosed in another hospital which was relieved by a percutaneous nephrostomy on the left side. Progressive deterioration at this point prompted referral to a tertiary center.

**Figure 1:**
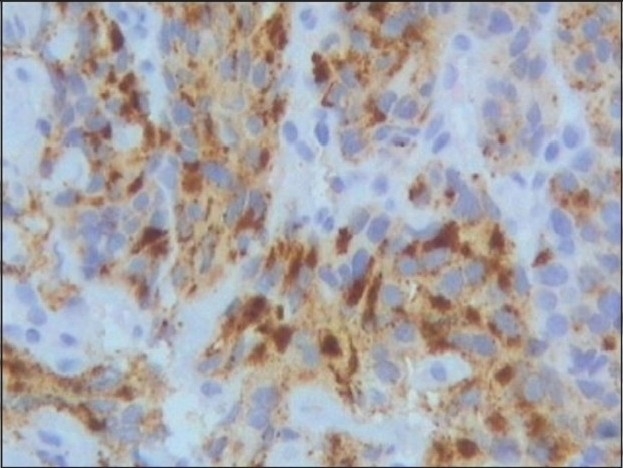
Photomicrograph of PSA immumohistochemical stain of peritoneal nodule core needle biopsy showing positive tumor cells. (40× magnification)

On evaluation, his serum PSA level was 82 ng/ml. Urethro-cystoscopy revealed a stricture of the prostato-membranous urethral junction and sub trigonal infiltration of the tumor in the bladder. MRI of the abdomen and pelvis showed extensive nodular deposits of intermediate intensity lining the peritoneum and omentum from the dome of the diaphragm to the pelvis. Bilateral mild hydroureteronephrosis and massive ascites were also seen. USG guided core needle biopsy of the peritoneal seedlings was done which showed sheets of undifferentiated carcinomatous cells. Ascitic fluid cytology showed a similar histological picture. A metastatic work-up to rule out another synchronous primary malignancy was undertaken. Esophagastroduodenoscopy and colonoscopy were negative and neither the MRI abdomen nor the chest radiograph detected any other primary source. Serum CEA level was 1.5ng/ml. A Tc^99m^- methylene diphosphonate (MDP) bone scan did not reveal any skeletal metastasis. Prostatic origin of the peritoneal deposits was confirmed by a PSA immunohistochemistry stain that was positive for tumor cells [Figure 1].

A diagnosis of hormone refractory prostate cancer with peritoneal and omental metastasis and malignant ascites was made. Treatment options were discussed with the patient. The ascites which was refractory to standard medical therapy with diuretics and an optimistic trial of octreotide necessitated daily drainage to alleviate respiratory distress. The patient was started on docetaxel based cytotoxic chemotherapy with steroids at three weekly intervals. The patient developed granulocytopenia that responded well to filigrastim. After the second cycle of chemotherapy, the frequency of ascitic taps decreased and his ascites was manageable medically. His serum creatinine level also improved from a baseline of 2.9 to 1.5 mg/dl. He then opted to be discharged from hospital to continue therapy from home.

## DISCUSSION

The most frequent sites of metastatic prostate carcinoma are lymph nodes and bone. After lymph nodes, bones and lung, the next most common regions of spread of prostate cancer at autopsy are bladder, liver, and adrenal gland.[1] Abdominal cavity spread was documented by Hess *et al*.[2] in 3 of 316 metastatic cancer prostate patients mostly in conjunction with skeletal or nodal spread.

Mechanisms of tumor spread have traditionally been explained by the mechanical theory of spread through lympho-vascular channels or alternatively through the “seed and soil” hypothesis. In this case, a direct spread from the bladder may be likely. Whether the peritoneum is ill-suited to sustain prostate tumor cells as opposed to bone and nodal tissues may only be known by experiments with suitable prostate cancer cell lines. Studying these rare occurrences may provide keys to unlock secrets of prostate cancer behaviour that may provide impetus to developing management strategies for the future. Turner *et al*, suggested that EGFR-mediated cell motility is an important mechanism involved in tumor progression, and that this cell property may represent a novel target to limit the spread of tumors.[3] PDGF, angiogenesis promoters (VEGF) and endothelin A are other targets being studied.

There are few reports of specific management initiatives for peritoneal disease in cancer prostate as it is an infrequent problem. Intraperitoneal inoculation of BCG suppressed propagation of prostatic adenocarcinoma - III cells (PA – III cell line) in the peritoneal cavity in rats.[4] Intraperitoneal instillation of chemotherapy has not been described for prostate cancer though it has been used in other tumors.

Peritoneal metastasis with ascites represents an advanced stage of an aggressive disease. Although a primary presentation may respond well to hormonal ablation therapy,[5] patients with hormone refractory disease may require careful evaluation for appraisal of the benefits and risks of palliation with potentially toxic agents. However, this should not deter treatment in patients who might have possible benefits or otherwise have limited options for palliation.
